# Association of the diplotype configuration at the *N*-acetyltransferase 2 gene with adverse events with co-trimoxazole in Japanese patients with systemic lupus erythematosus

**DOI:** 10.1186/ar2134

**Published:** 2007-03-03

**Authors:** Makoto Soejima, Tomoko Sugiura, Yasushi Kawaguchi, Manabu Kawamoto, Yasuhiro Katsumata, Kae Takagi, Ayako Nakajima, Tadayuki Mitamura, Akio Mimori, Masako Hara, Naoyuki Kamatani

**Affiliations:** 1 Institute of Rheumatology, Tokyo Women's Medical University School of Medicine, Kawada-cho, Shinjuku-ku, Tokyo 162-0054, Japan; 2 Department of Hematology and Rheumatology, JR Tokyo General Hospital, Yoyogi, Shibuya-ku, Tokyo, 151-8528, Japan; 3 Department of Rheumatology, International Medical Center of Japan, Toyama, Shinjuku-ku, Tokyo, 162-8855, Japan

## Abstract

Although co-trimoxazole (trimethoprim-sulphamethoxazole) is an effective drug for prophylaxis against and treatment of *Pneumocystis *pneumonia, patients often experience adverse events with this combination, even at prophylactic doses. With the aim being to achieve individual optimization of co-trimoxazole therapy in patients with systemic lupus erythematosus (SLE), we investigated genetic polymorphisms in the *NAT2 *gene (which encodes the metabolizing enzyme of sulphamethoxazole). Of 166 patients with SLE, 54 patients who were hospitalized and who received prophylactic doses of co-trimoxazole were included in the cohort study. Adverse events occurred in 18 patients; only two experienced severe adverse events that lead to discontinuation of the drug. These two patients and three additional ones with severe adverse events (from other institutions) were added to form a cohort sample and were analyzed in a case-control study. Genotype was determined using TaqMan methods, and haplotype was inferred using the maximum-likelihood method. In the cohort study, adverse events occurred more frequently in those without the NAT2*4 haplotype (5/7 [71.4%]) than in those with at least one NAT2*4 haplotype (13/47 [27.7%]; *P *= 0.034; relative risk = 2.58, 95% confidence interval = 1.34–4.99). In the case-control study the proportion of patients without NAT2*4 was significantly higher among those with severe adverse events (3/5 [60%]) than those without severe adverse events (6/52 [11.5%]; *P *= 0.024; odds ratio = 11.5, 95% confidence interval = 1.59–73.39). We conclude that lack of NAT2*4 haplotype is associated with adverse events with co-trimoxazole in Japanese patients with SLE.

## Introduction

Co-trimoxazole (trimethoprim-sulphamethoxazole) is an effective drug in the prevention and treatment of *Pneumocystis *pneumonia [1,2], a life-threatening condition that mainly occurs in immunodeficient patients. Usage of the drug was recently extended to patients with connective tissue disease, including systemic lupus erythematosus (SLE) [3]. Although co-trimoxazole was confirmed to have prophylactic effect against *Pneumocystis *pneumonia in SLE patients, it often causes adverse events, even at prophylactic doses. Adverse events include life-threatening conditions such as toxic epidermal necrolysis (TEN) and Stevens-Johnson syndrome (SJS), hepatotoxicity, haematological toxicity and gastrointestinal manifestations [4].

Of the two chemical components of co-trimoxazole, sulphamethoxazole is thought to be responsible for most cases of hypersensitivity [5]. The major metabolic pathway for sulphamethoxazole is catalyzed by *N*-acetylation by *N*-acetyltransferase 2 (NAT2). In the pathogenesis of hypersensitivity, the formation of hydroxylamine through oxidization by cytochrome P450 and its subsequent autooxidation to the nitroso metabolite have been implicated, although these are minor metabolic pathways [6-9]. These toxic metabolites are also detoxified by phase II enzymes and exhibit acetylation by NAT2, glucuronidation by uridine 5'-diphophate-glucronosyltransferase, sulphate conjugation by sulphotransferase, and conjugation with glutathione by glutathione *S*-transferase (GST) [7,10]. Among those metabolizing enzymes, NAT2 is a key enzyme because it catabolizes sulphamethoxazole and toxic metabolites, and may prevent the formation of hydroxylamine.

The *NAT2 *gene has at least 13 single nucleotide polymorphisms (SNPs) in the coding exon, and 29 NAT2 alleles (haplotypes) have been described in human populations, as shown in the NAT2 nomenclature Web site [11-13]. In addition to one wild-type haplotype (NAT2*4), the human *NAT2 *gene has four representative clusters of haplotypes that possess specific nucleotide substitutions at positions 341, 590, 857 and 191. These clusters are called NAT2*5, NAT2*6, NAT2*7 and NAT2*14, respectively. Previous studies have shown that the members of those clusters are responsible for the slow acetylator phenotype [14,15], which is conveniently determined by examining the concentration of caffeine in urine [16-18]. Individuals who are homozygous for mutant-type haplotypes exhibit the slow acetylator phenotype, whereas those who carry at least one wild-type haplotype exhibit the fast acetylator phenotype.

In the present study we examined the association between genetic polymorphisms in the *NAT2 *gene and adverse events with co-trimoxazole in patients with SLE.

## Materials and methods

### Patients and control individuals

The present study was approved by the Genome Ethics Committee of Tokyo Women's Medical University. A total of 166 patients with SLE were enrolled after they had given informed consent. Of these, 54 were admitted to our hospital between January 2001 and May 2006, and received co-trimoxazole (400 mg sulphamethoxazole and 80 mg trimethoprim) each day for prophylaxis against *Pneumocystis *pneumonia while they were immunosuppressed (CD4^+ ^cell count <200/mm^3^). All patients with SLE fulfilled the 1997 American College of Rheumatology revised criteria for the classification of SLE [19,20]. The data from these 54 patients were analyzed, and the patients were divided into two groups: those with adverse events (*n *= 18) and those without adverse events (*n *= 36). Among the 18 patients with adverse events, only two experienced severe events that lead to the discontinuation of co-trimoxazole treatment.

We collected samples from three additional patients at two other institutions who experienced severe adverse events. The five patients with severe adverse events (two from our institution and three from the other institutions) were combined to constitute a case group in a case-control study.

We wished to examine whether the genotypes or haplotypes of the *NAT2 *gene in SLE patients are different from those in patients with other connective tissue diseases or those from control subject individuals. We therefore obtained genomic DNA from 39 patients with polymyositis/dermatomyositis (PM/DM) who had fulfilled the criteria proposed by Bohan and Peter [21] and from 195 healthy donors (all gave informed consent). All patients and control individuals included in this study were Japanese.

### Assessment of adverse events with co-trimoxazole

Liver dysfunction was considered to be present when serum aspartate aminotransferase (AST) or alanine aminotransferase (ALT) levels were higher than twice the upper limit of the normal range. The patients and control individuals had no history of alcohol abuse and were negative for hepatitis B surface antigen and anti-hepatitis C virus antibody. Thrombocytopenia was defined as a platelet count below 100,000/μl. Rashes were variable in severity: TEN was diagnosed when there was widespread epidermal necrolysis with the appearance of scalding (>30% of the body surface area), and epidermal necrolysis that involved less than 10% of the body was diagnosed as SJS.

### DNA isolation

On admission to the hospital peripheral blood (10 ml) was drawn from each patient into tubes containing EDTA as an anticoagulant. A standard phenol-chloroform extraction procedure was used to extract genomic DNA from the blood samples.

### Genotyping at the single nucleotide polymorphisms in the *NAT2 *gene and inference of haplotype combinations

The *NAT2 *gene has at least 13 SNPs. Genotyping at four SNP sites enabled us to infer the haplotypes and diplotype configurations for the majority of the Japanese individuals. The four SNP sites included a C to T substitution at nucleotide position 282 (rs1041983), a C to T substitution at nucleotide position 481 (rs1799929), a G to A substitution at nucleotide position 590 (rs1799930) and a G to A substitution at nucleotide position 857 (rs1799931). These four SNPs yield six haplotypes in the *NAT2 *gene in the Japanese population, namely NAT2*4, NAT2*5B, NAT2*5E, NAT2*6A, NAT2*7B and NAT2*13. Of these, NAT2*4 is the wild-type haplotype; the remaining haplotypes are mutant types. In the present study, individuals who were homozygous for mutant-type haplotypes were tentatively designated slow acetylators; those who carried at least one wild-type haplotype were designated fast acetylators. A predeveloped TaqMan kit (Applied Biosystems, Foster City, CA, USA) that contained a set of forward and reverse primers and fluorescent-labelled probes that hybridize either wild-type or mutant-type sequences was used to determine genotypes at the four SNP sites by allelic discrimination chemistry. Genotypes were determined at four SNP loci in the *NAT2 *gene. From the obtained genotype data, we inferred the diplotype configuration for each individual, using PENHAPLO software [22,23]. This program was designed to infer haplotypes for each individual with using the maximum-likelihood method based on the expectation maximization algorithm, assuming Hardy-Weinberg equilibrium for the population [24]. This method infers not only the frequencies of haplotypes in the population but also the distribution of diplotype configurations in each individual.

### Statistical analysis

Fisher's exact test was used to evaluate differences in the frequencies of the diplotype configurations corresponding to the slow acetylators (those without NAT2*4) between the two groups. Differences were considered to be statistically significant at *P *< 0.05. Relative risks were determined in the cohort study and odds ratios were calculated in the case-control study, with 95% confidence intervals. SAS software (SAS Institute, Cary, NC, USA) was used to compare the differences in ALT levels between two groups using the nonparametric Mann-Whitney U-test.

## Results

### Haplotypes and diplotype configurations at the *NAT2 *gene

Genotyping of the four SNP sites at the *NAT2 *gene was sufficient for inferrence of haplotypes or diplotype configurations, using the PENHAPLO program, and the diplotype configuration was concentrated on a single haplotype combination for each individual using this program. Table 1 shows numbers and frequencies of diplotype configurations at the *NAT2 *gene in 166 patients with SLE, 39 patients with PM/DM, and 195 healthy individuals. The percentages of fast acetylators were 89.2%, 89.7% and 91.8% among patients with SLE, patients with PM/DM and healthy individuals, respectively (corresponding percentages of slow acetylators were 10.8%, 10.3% and 8.2%). The percentages of fast and slow acetylators were not statistically different between the three groups (*P *= 0.66 by Fisher's exact test).

**Table 1:** Numbers and frequencies of the diplotype configurations at the *NAT2 *gene among patients with SLE, those with PM/DM and healthy individuals

Diplotype configuration	Acetylator phenotype	SLE (*n *= 166)	PM/DM (*n *= 39)	Healthy individuals (*n *= 195)
NAT2*4/*4	Fast	84	14	103
NAT2*4/*5B	Fast	2	0	0
NAT2*4/*5E	Fast	1	0	0
NAT2*4/*6A	Fast	43	13	51
NAT2*4/*7B	Fast	17	8	24
NAT2*4/*13	Fast	1	0	1
Fast acetylators		148 (89.2)	35 (89.7)	179 (91.8)

NAT2*5B/*5B	Slow	0	0	1
NAT2*5B/*6A	Slow	0	0	2
NAT2*5B/*7B	Slow	1	0	0
NAT2*6A/*6A	Slow	5	2	6
NAT2*6A/*7B	Slow	9	1	6
NAT2*7B/*7B	Slow	3	1	1
Slow acetylators		18 (10.8)	4 (10.3)	16 (8.2)

Values are expressed as number (%) of patients. NAT2, *N*-acetyltransferase 2; PM/DM, polymyositis/dermatomyositis; SLE, systemic lupus erythematosus.

### Incidence of adverse events with co-trimoxazole

The incidence of adverse events was investigated prospectively in 54 patients with SLE who were receiving prophylactic doses of co-trimoxazole. Adverse events occurred in 18 (33.3%) of the 54 patients. Of the 18 patients with SLE who experienced adverse events, two had dual adverse events (yielding a total of 20 adverse events). Of the 20 adverse events, 14 (70%) were liver dysfunction, five (25%) were thrombocytopenia and one (5%) was skin rash. None of fever, gastrointestinal symptom, headache, anaemia, or leucopaenia was observed in the study group.

### Association between diplotype configurations at the *NAT2 *gene and adverse events with co-trimoxazole

The association between diplotype configurations at the *NAT2 *gene and occurrence of adverse events with co-trimoxazole in the cohort study is shown in Table 2. Five out of seven (71.4%) slow acetylators experienced adverse events, and the frequency was significantly higher than that among fast acetylators (13/47 [27.7%]; *P *= 0.034 by Fisher's exact test; relative risk = 2.58, 95% confidence interval = 1.34–4.99). Frequency of immunosuppressant use did not differ between patients who suffered adverse events with co-trimoxazole and those who did not. Thus, concomitant medications did not appear to influence the occurrence of adverse events of co-trimoxazole.

**Table 2:** Association between diplotype configurations at the *NAT2 *gene and adverse events with co-trimoxazole, analyzed in the cohort study

Diplotype configuration	Acetylator phenotype	With adverse events (*n *= 18)	Without adverse events (*n *= 36)	Total
NAT2*4/*4	Fast	6	15	21
NAT2*4/*5B	Fast	0	2	2
NAT2*4/*5E	Fast	0	1	1
NAT2*4/*6A	Fast	5	12	17
NAT2*4/*7B	Fast	2	4	6
Fast acetylators		13 (27.7)	34 (72.3)	47

NAT2*6A/*6A	Slow	1	0	1
NAT2*6A/*7B	Slow	3	1	4
NAT2*7B/*7B	Slow	1	1	2
Slow acetylators		5 (71.4)^a^	2 (28.6)	7

Values are expressed as number (%) of patients. The frequency of adverse events was compared between fast acetylators (*n *= 47) and slow acetylators (*n *= 7). ^a^*P *= 0.034 versus fast acetylators (by Fisher's exact test); relative risk = 2.58, 95% confidence interval = 1.34–4.99. NAT2, *N*-acetyltransferase 2.

Five patients with severe adverse events were analyzed in a case-control study. Severe adverse events included TEN, SJS, severe liver dysfunction (ALT >300 IU/ml) and severe thrombocytopenia (platelets <50,000/μl), and are summarized in Table 3. As shown in Table 4, three of the five patients with severe adverse events were slow acetylators, and the frequency was compared with that among 52 SLE patients who did not experience severe adverse events (including 16 patients with mild adverse events and 36 patients with no adverse events). The poportion of slow acetylators was significantly higher among the patients with severe adverse events than in those without (60% versus 11.5%; *P *= 0.024 by Fisher's exact test, odds ratio = 11.5, 95% confidence interval = 1.59–73.39).

**Table 3:** Clinical characteristics and diplotype configurations at the *NAT2 *gene in five patients who experienced severe adverse events with co-trimoxazole

Patient number	Age (years)/sex	Diplotype configuration	Acetylator phenotype	Adverse events
1	52/female	NAT2*6A/*6A	Slow	TEN, liver dysfunction
2	56/female	NAT2*5B/*7B	Slow	SJS, liver dysfunction
3	43/female	NAT2*6A/*7B	Slow	Thrombocytopenia, liver dysfunction
4	48/female	NAT2*4/*7B	Fast	Thrombocytopenia
5	46/female	NAT2*4/*6A	Fast	SJS, liver dysfunction

NAT2, *N-*acetyltransferase 2; SJS, Stevens-Johnson syndrome; TEN, toxic epidermal necrolysis.

**Table 4:** Association between diplotype configurations at the *NAT2 *gene and severe adverse events with co-trimoxazole, analyzed in the case-control study

Diplotype configuration	With severe adverse events (*n *= 5)	Without severe adverse events (*n *= 52)
Slow acetylator (without NAT2*4)	3 (60)^a^	6 (11.5)
Fast acetylator (with NAT2*4)	2 (40)	46 (88.5)

Values are expressed as number (%) of patients. The frequency of slow acetylators was compared between five patients who experienced severe adverse events and 52 patients without severe adverse events. ^a^*P *= 0.024 by Fisher's exact test; odds ratio = 11.5, 95% confidence interval = 1.59–73.39. NAT2, *N*-acetyltransferase 2.

### Influence of diplotype configurations at the *NAT2 *gene on serum markers of liver dysfunction

**Figure 1:**
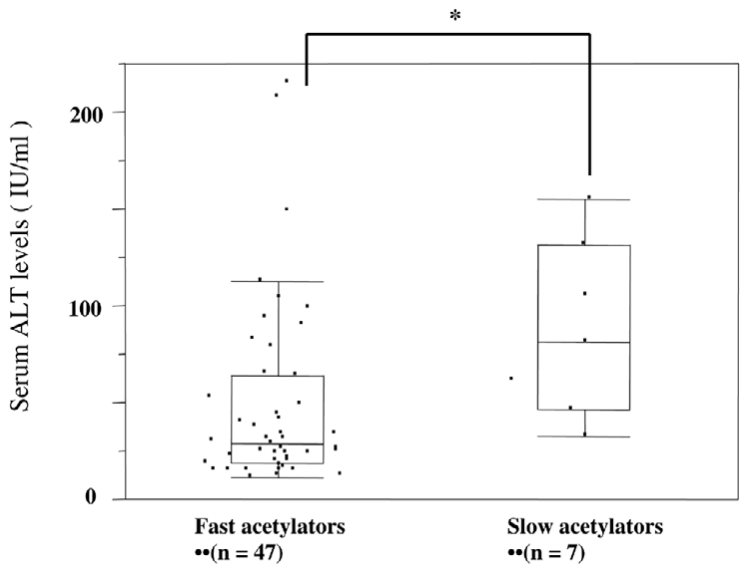
Levels of serum ALT in fast acetylators and slow acetylators. There were 47 fast acetylators and seven slow acetylators in the cohort. Levels of serum ALT in patients with systemic lupus erythematosus were measured 14 day after initiation of co-trimoxazole. Serum ALT levels in slow acetylators were significantly higher than in fast acetylators (median: 82.0 ± 45.3 IU/ml versus 30.0 ± 47.0 IU/ml; *P = 0.0096, by Mann-Whitney U-test). ALT, alanine aminotransferase.

Liver dysfunction as an adverse event can be statistically evaluated using serum markers (ALT and AST). The mean (± standard deviation) interval between the initiation of co-trimoxazole treatment and first observation of liver dysfunction was 15.8 ± 5.2 days. Figure 1 shows serum ALT levels on day 14 after initiation of treatment with co-trimoxazole. Levels of serum ALT were significantly higher in slow acetylators than in fast acetylators (median: 82.0 ± 45.3 IU/ml versus 30.0 ± 47.0 IU/ml; *P *= 0.0096 by Mann-Whitney U-test). Serum AST levels immediately after the administration of co-trimoxazole were not significantly different between fast and slow acetylators (median: 30.0 ± 21.5 IU/ml versus 24.0 ± 31.0 IU/ml; *P *= 0.088 by Mann-Whitney U-test). In both groups, baseline levels of serum AST and ALT before administration of co-trimoxazole were within normal limits.

## Discussion

The present study demonstrates that, among patients with SLE, slow acetylators (as inferred based on genotype data in the *NAT2 *gene) exhibit a greater frequency of various adverse events with co-trimoxazole than do fast acetylators. Ohno and coworkers [14] first reported an association between genetic polymorphisms at the *NAT2 *gene and the occurrence of adverse events with sulphonamides. Since then severe adverse events with sulfasalazine, another compound that is catabolized by NAT2, were also reported to be associated with absence of the wild-type allele (NAT2*4) in the *NAT2 *gene [15,25]. There have been conflicting reports regarding the association between adverse events with co-trimoxazole and *NAT2 *genotype, with findings apparently correlating with the underlying disease process. For instance, in the setting of HIV infection many investigators were unable to demonstrate such an association, but some reports have shown a positive association in patients without HIV infection [26]. Therefore, differences in background illness may account for the fact that a positive association was observed in the present study (in patients with SLE) and negative associations were observed in other reports in which the disease was HIV related. An alternative explanation is that there are differences in composition of *NAT2 *haplotypes between ethnic groups (as discussed below).

In the present study we found that slow acetylators at the *NAT2 *gene, among the patients with SLE, were more likely to develop adverse events with co-trimoxazole than were fast acetylators in the cohort study (relative risk = 2.58). In the case-control study, we found that the proportion of slow acetylators was higher among patients with severe adverse events than in those without (60% versus 11.5%; odds ratio = 11.5), although we could not find statistically significant differences between the patients with severe adverse events and 16 patients with mild adverse events (60% versus 25%; *P *= 0.28). We emphasize that severe adverse events, including life-threatening ones, were associated with *NAT2 *polymorphisms.

The most frequent adverse event with co-trimoxazole in the cohort study group was liver dysfunction (70%), followed by thrombocytopenia (25%) and rash (5%). The types of adverse events are slightly different from those previously reported for co-trimoxazole. Karpman and Kurzrock [27] reviewed the adverse events associated with co-trimoxazole use in children on full-dose therapy, and indicated that cutaneous lesions were the most common hypersensitivity reactions to co-trimoxazole, accounting for 70% of all adverse events. Other hypersensitivity effects, including fever and haematological toxicity, were also frequent. Liver dysfunction was less common. With sulphonamides, however, liver dysfunction is a well documented common adverse event [28]. We speculate that the incidence of liver dysfunction in patients receiving co-trimoxazole might have been underestimated in the study by Karpman and Kurzrock [27]. All patients in the present study were hospitalized and monitored for asymptomatic liver dysfunction using routine biochemical tests. Among the 54 patients with SLE who were enrolled in the cohort study, slow acetylators exhibited significantly higher levels of serum ALT after administration of co-trimoxazole than did fast acetylators. This suggests that *NAT2 *genotype affected levels of serum ALT. On the other hand, the low incidence of cutaneous lesions in the present cohort study might have been the result of preceding immunosuppressive therapies, latently preventing development of cutaneous lesions.

Sulphonamides are the compounds most associated with development of TEN, and the mechanism of skin necrolysis is reported to be through cytotoxic lymphocyte-mediated pathways and clonally expanded CD8^+ ^T cells [29]. Although TEN and SJS induced by co-trimoxazole is rare, it is a major problem because severe skin disease such as TEN and SJS can occur even with prophylactic doses of co-trimoxazole in SLE. Indeed, some patients in the case-control study exhibited hypersensitivity reactions, including TEN and SJS, which are typical and severe allergic reactions to the drug. Interestingly, a large proportion of these patients were slow acetylators. Thus, polymorphisms at the *NAT2 *gene may account even for allergic reactions to co-trimoxazole. These findings are consistent with a previous report [15] demonstrating that allergic reactions such as rashes and fever were more frequent in slow acetylators (as inferred by the maximum-likelihood method) among Japanese patients with rheumatoid arthritis treated with sulphasalazine. The greater frequency of adverse events with co-trimoxazole in slow acetylators might be accounted for by delayed drug clearance, resulting in sustained high levels of serum sulphamethoxazole, which is likely to lead to increased formation of hydroxylamine or nitroso-sulphamethoxazole. These toxic metabolites and sulphamethoxazole might act as cytotoxic, genotoxic, or immunogenic agents, and hence induce adverse reactions.

Haplotype frequencies at the *NAT2 *gene vary between ethnic groups. The percentage of slow acetylators was reported to be 56% to 74% among Caucasians [30], but it was only 8.2% in our study. The percentage of slow acetylators in our study is similar to proportions reported previously in the Japanese population [15]. Furthermore, the compositions of mutant haplotypes are quite different between Caucasian and Japanese individuals. In the former, the most frequent mutant haplotype is the NAT2*5 cluster (45%), followed by the NAT2*6 cluster (28%) and the NAT2*7 cluster (2%) [31]. In the Japanese population, however, NAT2*6A is the most frequent (20%), followed by NAT2*7B (13%), and the NAT2*5 cluster is very rare (0.01%). Thus, major components of the *NAT2 *mutant haplotype in Caucasian individuals are NAT2*5B and NAT2*6A; in the Japanese population, NAT2*6A and NAT2*7B are the major components. Each haplotype contains specific nucleotide substitutions: T341C and A803G for NAT2*5B, G590A for NAT2*6A, and G857A for NAT2*7B. All of these substitutions cause amino acid changes. It is curious, however, that the severe adverse events caused by sulfasalazine, associated with *NAT2 *variations, have been reported exclusively from Japan, although the frequency of slow acetylators is much higher among Caucasian than Japanese populations [15,25]. The quiet different composition of mutant haplotypes between Caucasian and Japanese populations may account for the difference in adverse events relative to *NAT2 *gene haplotype.

During metabolic detoxification of sulphamethoxazole, phase II enzymes other than NAT2 also play a role. GST can detoxify nitroso-sulphamethoxazole by reduction back to hydroxylamine. It catalyzes conjugation of electrophiles with glutathione, thereby inactivating those often cytotoxic or genotoxic substances [32]. Of several GST isozymes, the μ-class enzyme (GSTM), the θ-class enzyme (GSTT) and the π-class enzyme (GSTP) are polymorphic [33-36]. The genetic polymorphisms at the *GSTM1 *and *GSTT1 *genes were reported to be deletion of nucleotides (referred to as null *GSTM1 *and null *GSTT1 *alleles), which have been associated with susceptibility to cancer [37,38]. We also investigated the involvement of genetic polymorphisms of the *GSTT1 *and *GSTM1 *genes in the occurrence of adverse events with co-trimoxazole (data not shown). Nevertheless, none of the *GST *gene polymorphisms were associated with adverse events, a finding that is consistent with previous data [30].

## Conclusion

We found that Japanese patients with SLE who do not harbour the NAT2*4 haplotype develop adverse events with co-trimoxazole more frequently than patients with at least one NAT2*4 haplotype. Knowledge on diplotype configurations for the *NAT2 *gene may lead to improveed efficacy and safety of co-trimoxazole in patients with SLE.

## Abbreviations

ALT = alanine aminotransferase; AST = aspartate aminotransferase; GST = glutathione *S*-transferase; NAT2 = *N*-acetyltransferase 2; PM/DM = polymyositis/dermatomyositis; SLE = systemic lupus erythematosus; SNP = single nucleotide polymorphism; SJS = Stevens-Johnson syndrome; TEN = toxic epidermal necrolysis.

## Competing interests

The authors declare that they have no competing interests.

## Authors' contributions

MS conceived the study and drafted the manuscript. TS, together with Y Kawaguchi, participated in the design and coordination of the study. MK was responsible for using PENHAPLO software. Y Katsumata, KT, AN, TM and AM recruited a subset of patients. MH recruited a subset of patients and participated in coordination of the study. NK participated in the design and coordination of the study. All authors read and approved the final manuscript.
